# Severe pulmonary tuberculosis complicating Ileocecal intussusception due to intestinal tuberculosis: a case report

**DOI:** 10.1186/1476-0711-7-16

**Published:** 2008-07-13

**Authors:** Shigeki Nakamura, Katsunori Yanagihara, Koichi Izumikawa, Masafumi Seki, Hiroshi Kakeya, Yoshihiro Yamamoto, Yoshitugu Miyazaki, Naofumi Suyama, Shigeru Kohno

**Affiliations:** 1 Second Department of Internal Medicine, Nagasaki University Graduate School of Medical Science, Nagasaki, Japan; 2 Department of Laboratory Medicine, Nagasaki University Graduate School of Medical Science, Nagasaki, Japan; 3 Nagasaki Municipal Medical Center, Nagasaki, Japan; 4 Division of Molecular & Clinical Microbiology, Department of Molecular Microbiology & Immunology, Nagasaki University Graduate School of Medical Science, Nagasaki, Japan

## Abstract

Adult intussusception is a rare clinical entity that is most often caused by a tumor, such as a lipoma, adenoma, or malignant tumor. A case of adult intussusception due to intestinal tuberculosis of the ileocecal region is reported. There are few cases of intussusception due to intestinal tuberculosis.

## Background

Intestinal tuberculosis continues to be a major problem in many regions of the world. In the abdomen, tuberculosis may affect the intestinal tract, lymph nodes, and peritoneum. The lack of specific signs and symptoms of abdominal tuberculosis involving the intestinal tract frequently leads to missed or delayed diagnoses, which can result in severe complications that are associated with intestinal tuberculosis, including obstruction, perforation, and fistula formation [1], though intussusception is uncommon. The case of a 76-year-old female who developed intussusception due to intestinal tuberculosis is reported. Adult intussusception is rare, and few cases caused by intestinal tuberculosis have been reported. Generally, a surgical procedure is needed for adult intussusception, and the outcome is often poor [2,3]. Early and proper diagnosis of intestinal tuberculosis is necessary to prevent severe complications.

## Case report

**Figure 1:**
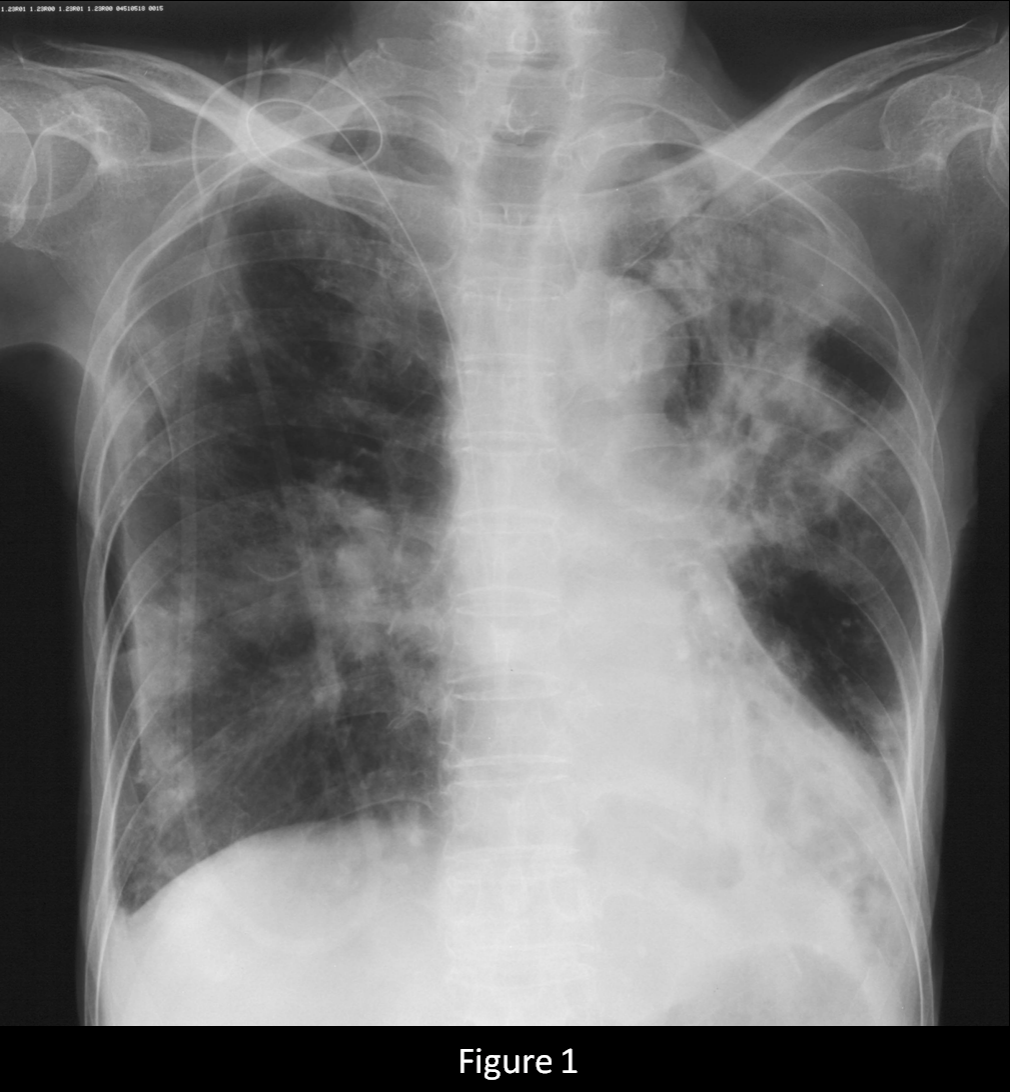
Chest radiograph showing consolidation in both lung fields and a cavity in the left upper lung field.

**Figure 2:**
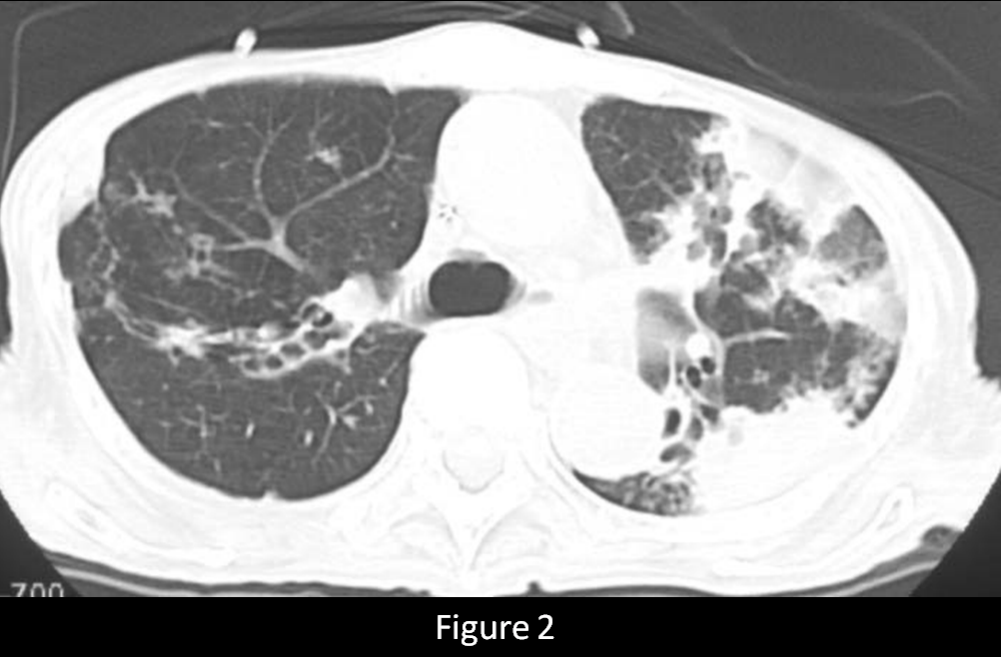
Chest computed tomography scan showing multiple consolidations in both lung and a cavity in the left upper lobe.

**Figure 3:**
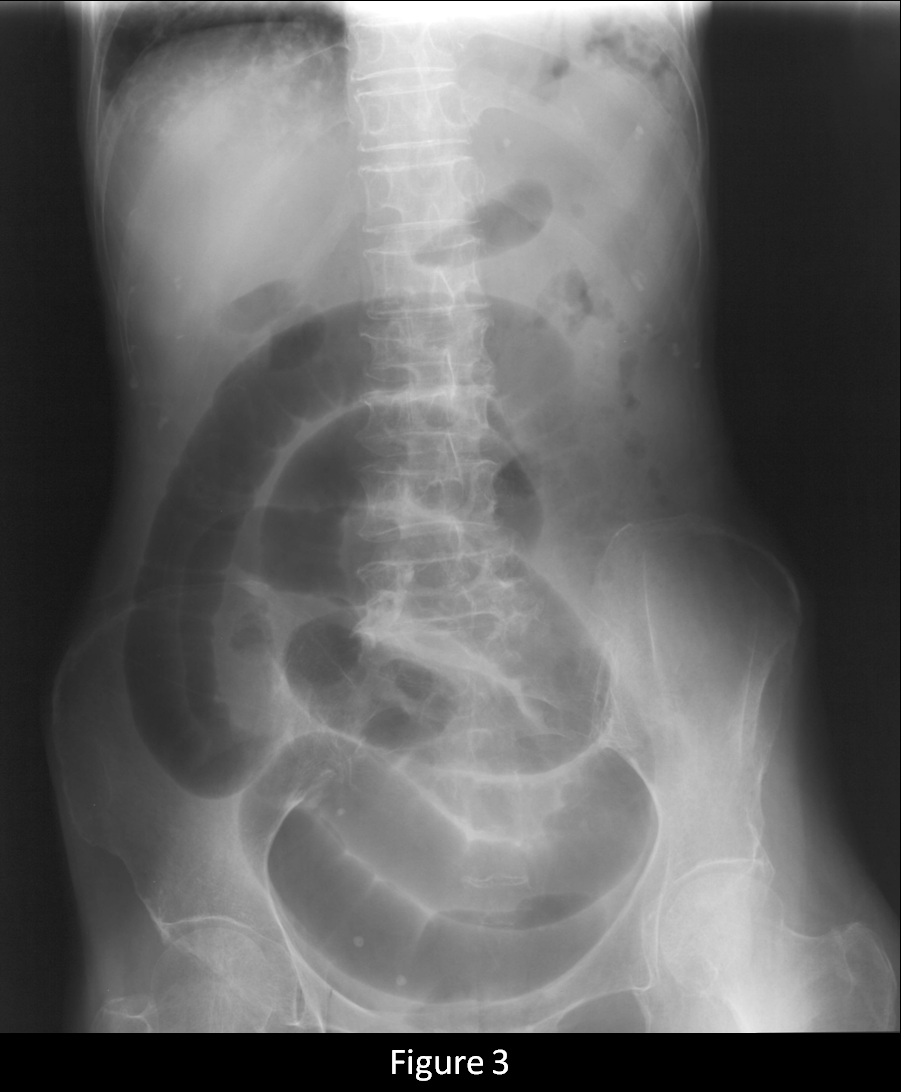
Abdominal radiograph showing a markedly dilated small intestine.

**Figure 4:**
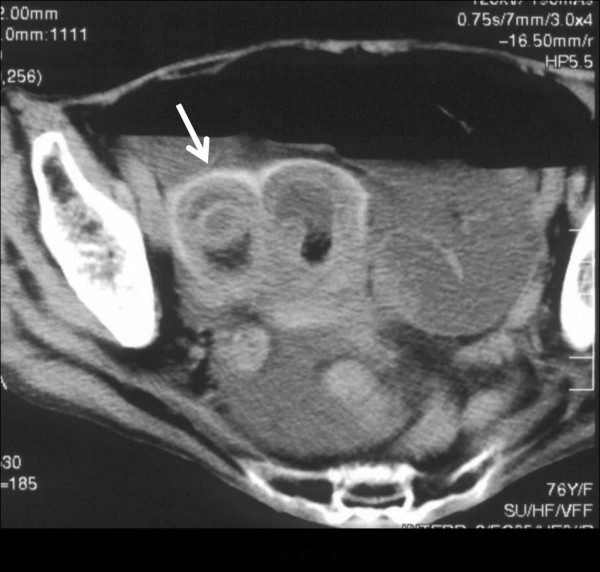
Abdominal computed tomography scan showing an inhomogenous soft tissue mass that is target- or sausage-shaped in an ileo-colonic intussusception caused by intestinal tuberculosis (Arrow).

**Figure 5:**
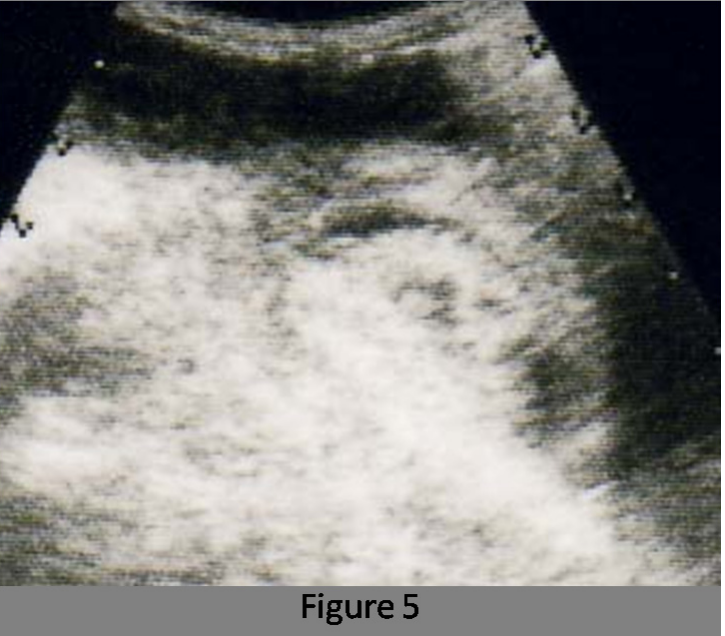
The target sign is evident in the ileocecal region with alternating hypoechoic and hyperechoic layers and a hyperechogenic center.

**Figure 6:**
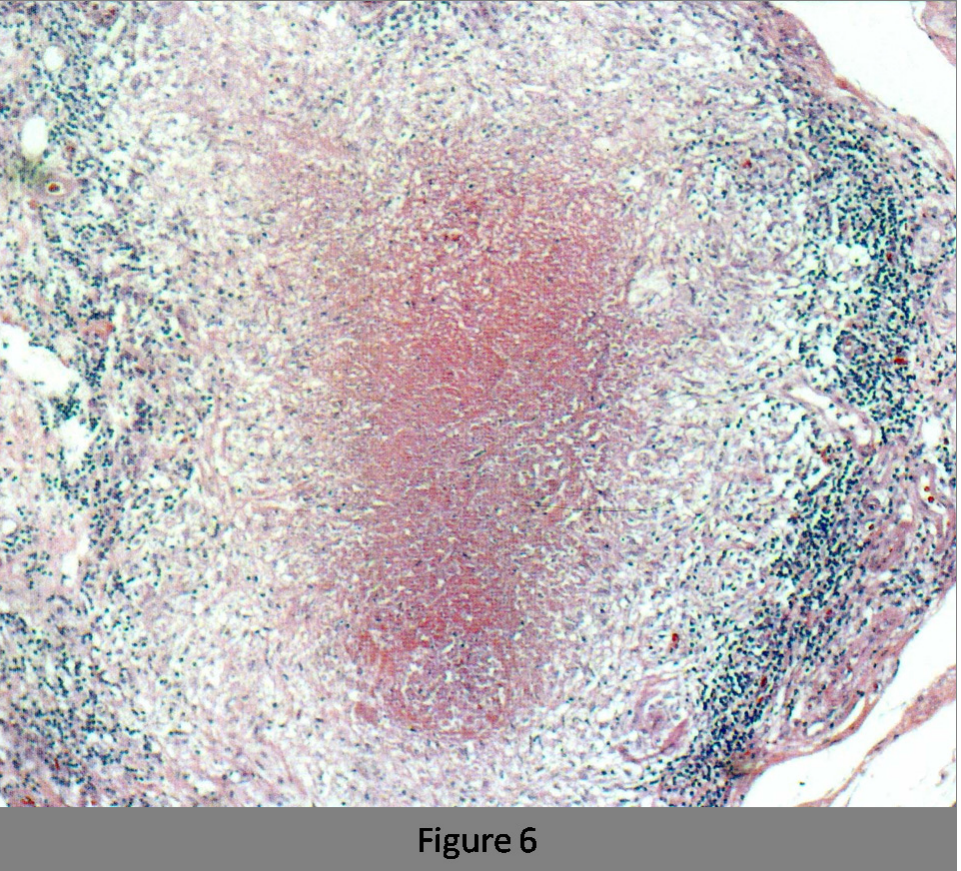
Granulomatous structure and caseous necrosis in the intestinal mucosa.

A 76-year-old female who had never smoked came to the hospital because of chronic cough. The chest radiograph showed bilateral consolidation with a cavity (Figure 1). The chest computed tomography (CT) scan showed multiple consolidations in both lungs with cavities in the left upper lobe (Figure 2). The acid-fast stain of the sputum smear was positive, and the sputum culture and the PCR were also positive for *M. tuberculosis*. Thus, the patient was diagnosed as having pulmonary tuberculosis, and she was admitted to hospital for treatment. Three days later from admission to prior hospital, the patient developed acute abdominal pain with guarding. An abdominal radiograph showed marked dilatation of the small intestine (Figure 3). On enhanced abdominal CT (Figure 4), intestinal obstruction caused by a mass lesion with a target sign in the ascending colon was seen. A concentric ring of intraluminal mesenteric fat interposed between the central portion and the outer edematous colonic wall gives this lesion its characteristic appearance. In addition, the small intestine was enlarged and filled with fluid. The patient was diagnosed as having intussusception and was admitted to our hospital to treat both the pulmonary tuberculosis and the intussusception. On physical examination, her body temperature was 37.8°C, the heart rate was 86 beats/min, and the respiratory rate was 18 breaths/min. Her blood pressure was 150/72 mmHg. Coarse crackles were audible in the left lung. The patient had no peripheral lymphadenopathy, no skin lesions, and no neurological deficits. On abdominal examination, the mass lesion was palpable in the right lower abdominal region, and guarding was present. On laboratory examination, the white blood cell count was elevated (15.4 × 10^3^/mm^3^), the serum C-reactive protein (CRP) level was increased (5.43 mg/dl), and the erythrocyte sedimentation rate was increased (70 mm/h). Other laboratory results included: total protein, 5.9 g/dl; serum albumin, 3.4 g/dl; serum creatinine, 0.5 mg/dl; blood urea nitrogen, 14.0 mg/dl; and serum lactate dehydrogenase, 301 IU/l. The serum glucose concentration and liver function tests were normal, and the serum complement prothrombin time was 97%. The HIV antibody was negative and the numbet of CD4 positive T-lymphocyte was normal. Arterial blood gases were improved on 2L O_2 _given nasally (PaO_2_, 80.1 Torr; PaCO_2_, 52.1 Torr; pH, 7.46; HCO_3_, 36.5 mmol/L; BE 11.3 mmol/L). Abdominal ultrasonography showed the bull's eye sign at the site of the mass lesion (Figure 5). Since there was a possibility of intestinal necrosis, ileocecal resection was done on the day of admission. Macroscopically, it could be seen that the terminal ileum gained entry to the ascending colon. Pathologically, there were large areas of ischemic enteritis due to intussusceptions, and caseous necrosis with epithelioid cell granulomas was seen (Figure 6). There was no evidence of any neoplasm or diverticulum that could have caused the intussusception. Thus, this patient was diagnosed as having intussusception due to intestinal tuberculosis. The patient was treated with isoniazid (300 mg/day), rifampicin (600 mg/day), ethambutol (750 mg/day), and pyrazinamide (750 mg/day). The patient's postoperative course was uneventful, and there has been no recurrence.

## Discussion

Intussusception occurs when a proximal segment of bowel and its associated mesentery telescope into the lumen of the adjacent distal segment. It is a rare entity in adults; it accounts for only 1% of all cases of intestinal obstruction and 5% of all intussusceptions [4,5]. Intussusceptions involving the small intestine are, in about 90% of cases, the result of benign processes, including polyps, such as lipomas, adenomas, and inflammatory polyps, Meckel's diverticulum, and adhesions [6-8]. On the other hand, in the large intestine, malignant processes, usually adenocarcinomas, are common causes of intussusception [9]. Abdominal tuberculosis is still a major problem in many regions of the world. In particular, the incidence of intestinal tuberculosis has been increasing in the West, due to the AIDS epidemic, transglobal migration, an aging population, and an increasing number of immunosuppressed patients [10,11]. The abdominal complications of intestinal tuberculosis include perforation, fistula formation, and intestinal bleeding [1]. Obstruction is the most common complication, and it occurs in 12–60% of cases [12]. Intussusception is a very rare complication. In the present case, the inflammatory lesions formed by the advanced intestinal tuberculosis likely caused the intussusception. Intestinal tuberculosis is not commonly diagnosed early because the symptoms are often non-specific. It has been reported that abdominal CT findings can help in making the diagnosis of intestinal tuberculosis [11]. When the inflammatory process is mild, CT shows only slight and symmetric wall thickening and a few small regional lymph nodes, while in the advanced stage, CT shows asymmetric thickening of the ileocecal valve and medial wall of the cecum, and a heterogeneous soft-tissue mass that envelopes the terminal ileum. The location of the disease is also helpful in making the diagnosis of intestinal tuberculosis. Approximately 75% of intestinal tuberculosis patients have involvement of the distal small bowel and ileocecal region [13]. In addition, abdominal CT has been reported to be the most useful tool for diagnosis of adult intussusception, with a diagnostic accuracy rate of 58–100% [14,15]. In the present case, the patient likely had intestinal tuberculosis for a long time, and it was found because of the development of the intussusception. Furthermore, some authors have reported that patients with diseases causing immunosuppression are more likely to have severe intestinal tuberculosis [16,17]. Thus, the present patient probably developed intestinal tuberculosis due to her immunosuppressed status as a result of severe undernutrition and advanced age.

## Conclusion

Intussusception is rare in the adult population, and, to the best of our knowledge, intussusception caused by intestinal tuberculosis is very rare. Early detection of intestinal tuberculosis is important to avoid catastrophic events, including intussusception, since emergency surgery for such lesions carries a high mortality rate. Abdominal CT appears to be helpful in making an early diagnosis and preventing the severe complications of intestinal tuberculosis.

## Consent

We did informed consent for the patient about publishing the manuscript to *Annals of Clinical Microbiology and Antimicrobials*. Written informed consent was obtained from the patient for publication of this case report and any accompanying images. A copy of the written consent is available for review by the Editor-in-Chief of this journal.

## Competing interests

We don't have any financial relationship with other people or organizations. And we don't have any financial competing interests that may cause them embarrassment were they to become public after the publication of the manuscript.

The all authors confirm that this manuscript is original and has not been submitted elsewhere.

And all authors confirm that there are any non-financial competing interests to declare in relation to this manuscript.

## Authors' contributions

Each author acknowledges that he has contributed in a substantial way to the work described in the manuscript and its preparation. All authors read and approved the final manuscript.
